# EhNPC1 and EhNPC2 Proteins Participate in Trafficking of Exogenous Cholesterol in *Entamoeba histolytica* Trophozoites: Relevance for Phagocytosis

**DOI:** 10.1371/journal.ppat.1006089

**Published:** 2016-12-21

**Authors:** Jeni Bolaños, Abigail Betanzos, Rosario Javier-Reyna, Guillermina García- Rivera, Miriam Huerta, Jonnatan Pais-Morales, Arturo González-Robles, Mario A. Rodríguez, Michael Schnoor, Esther Orozco

**Affiliations:** 1 Departamento de Infectómica y Patogénesis Molecular, Centro de Investigación y de Estudios Avanzados del Instituto Politécnico Nacional, D.F., México; 2 Cátedras, Consejo Nacional de Ciencia y Tecnología, D.F., México; 3 Departamento de Biomedicina Molecular, Centro de Investigación y de Estudios Avanzados del Instituto Politécnico Nacional, D.F., México; University of Virginia Health System, UNITED STATES

## Abstract

*Entamoeba histolytica*, the highly phagocytic protozoan causative of human amoebiasis lacks the machinery to synthesize cholesterol. Here, we investigated the presence of NPC1 and NPC2 proteins in this parasite, which are involved in cholesterol trafficking in mammals. Bioinformatics analysis revealed one *Ehnpc1* and two *Ehnpc2* genes. EhNPC1 appeared as a transmembrane protein and both EhNPC2 as peripheral membrane proteins. Molecular docking predicted that EhNPC1 and EhNPC2 bind cholesterol and interact with each other. Genes and proteins were identified in trophozoites. Serum pulse-chase and confocal microscopy assays unveiled that after trophozoites sensed the cholesterol source, EhNPC1 and EhNPC2 were organized around the plasma membrane in a punctuated pattern. Vesicles emerged and increased in number and size and some appeared full of cholesterol with EhNPC1 or EhNPC2 facing the extracellular space. Both proteins, but mostly EhNPC2, were found out of the cell associated with cholesterol. EhNPC1 and cholesterol formed networks from the plasma membrane to the nucleus. EhNPC2 appeared in erythrocytes that were being ingested by trophozoites, co-localizing with cholesterol of erythrocytes, whereas EhNPC1 surrounded the phagocytic cup. EhNPC1 and EhNPC2 co-localized with EhSERCA in the endoplasmic reticulum and with lysobisphosphatidic acid and EhADH (an Alix protein) in phagolysosomes. Immunoprecipitation assays confirmed the EhNPC1 and EhNPC2 association with cholesterol, EhRab7A and EhADH. Serum starved and blockage of cholesterol trafficking caused a low rate of phagocytosis and incapability of trophozoites to produce damage in the mouse colon. *Ehnpc1* and *Ehnpc2* knockdown provoked in trophozoites a lower intracellular cholesterol concentration and a diminished rate of phagocytosis; and *Ehnpc1* silencing also produced a decrease of trophozoites movement. Trafficking of EhNPC1 and EhNPC2 during cholesterol uptake and phagocytosis as well as their association with molecules involved in endocytosis strongly suggest that these proteins play a key role in cholesterol uptake.

## Author Summary

NPC1 and NPC2 proteins are involved in cholesterol trafficking in mammals. Using different approaches, we have detected the orthologues EhNPC1 and EhNPC2 proteins in *Entamoeba histolytica*. Trophozoites are particularly rich in membranes and vacuoles, but they do not possess the machinery to synthetize cholesterol. Thus, they are completely dependent on molecules able to “fish” cholesterol from the medium. The relevance of our findings lies in the fact that cholesterol is fundamental for endocytosis and motility; and, phagocytosis is an important nutritional and virulence factor for *E*. *histolytica*. *In silico* and experimental strategies, using U18666A to arrest cholesterol trafficking, as well as, knockdown mutants, showed that EhNPC1 and EhNPC2 participate in cholesterol uptake and trafficking in this parasite. They are secreted by trophozoites and directly involved in erythrophagocytosis and motility. Our findings revealed *E*. *histolytica* as one of the first protozoa in which these proteins are being characterized. Moreover, *E*. *histolytica* provides an excellent and less complicated model to elucidate the intricate event of cholesterol trafficking in eukaryotic cells. The relevance of cholesterol transport for the parasite virulence and the involvement of EhNPC1 and EhNPC2 in this process, make these proteins promising targets for therapy strategies development against the parasite.

## Introduction

*Entamoeba histolytica* is the protozoan responsible for intestinal and hepatic amoebiasis, considered the third leading cause of death worldwide due to parasites [1]. *E*. *histolytica* trophozoites are highly dynamic cells with active movement and voracious phagocytosis. High cholesterol concentration in the medium enhances their virulence [2–4] and trophozoites loaded with cholesterol showed an enrichment of the Gal/GalNAc lectin in rafts and an increase of amoeba adherence to target cells [5]. Cholesterol is fundamental for vesicle formation and lipid rafts arrangements, and both are crucial events for movement and endocytosis [4]. However, *E*. *histolytica* lacks the machinery to synthesize cholesterol [6]. Cells ingested by the parasite, including erythrocytes, are a natural cholesterol source, but, trophozoites can also uptake it from the serum-supplemented culture medium [7, 8].

Mammalian cells synthesize cholesterol through a complex pathway, in which at least 30 enzymes participate [9]. Cholesterol homeostasis is controlled by feedback regulation of its biosynthesis and uptake through receptor-mediated endocytosis by low density lipoproteins (LDL) [10]. Failures in cholesterol storage in humans cause the Niemann-Pick type C (NPC) disease, which is linked to mutations in NPC1 or NPC2 proteins that are directly involved in cholesterol trafficking [11, 12]. NPC1 (1278 amino acids) is a polytopic endosomal membrane glycoprotein required for efflux of cholesterol from endosomes [13]. It has 13 transmembrane domains, four luminal and six cytoplasmic loops, a C-terminal cytoplasmic tail and a sterol sensing domain (SSD) [14]. NPC2 (151 amino acids) is a soluble lysosomal protein with a MLD domain (ML [MD-2 (myeloid differentiation factor-2)]-related lipid-recognition) [15, 16] that regulates cholesterol trafficking from lysosomes to the endoplasmic reticulum (ER) [17, 18]. NPC2 possesses positively charged regions that facilitate its interaction with negatively charged membranes [17]. NPC2 binds to NPC1; and NPC1 binds to cholesterol by the SSD domain in an acidic milieu [18]. Moreover, cholesterol is transferred from the N terminus domain (NTD) of NPC1 to NPC2 in a bidirectional manner [19]. Based on this, Infante *et al*. [19] proposed the “hand-off” working model, which assumes that NPC2 takes cholesterol in the lysosomal lumen and transports it to membrane-bound NPC1 for exportation to the ER [19–21]. In late endosomes, lysobisphosphatidic acid (LBPA) regulates cholesterol, under the control of Alix protein [22, 23]. The molecular mechanisms of this regulation are not completely understood.

In *E*. *histolytica*, neither LDL receptors, nor NPC1 and NPC2 have been identified yet. The TMK39 protein participates in cholesterol uptake, however, it does not have cholesterol binding domains, suggesting that it could associate to cholesterol through other trophozoite molecules [24]. As in mammals, *E*. *histolytica* LBPA binds to EhADH (an *E*. *histolytica* ALIX family protein) [25] inside phagolysosomes and multivesicular bodies (MVB) [26]. Though, we do not discard the participation of other Alix proteins involved in endomembrane trafficking [27, 28]. Thus, it is plausible to assume that the “hand-off” model, “NPC2-NPC1-cholesterol-NPC2-NPC1” proposed for mammalian cells, could also be functioning in trophozoites to first carry exogenous cholesterol inside the cell and then, to transport it to distinct organelles. Here, we searched for NPC1 and NPC2 orthologues in *E*. *histolytica* and studied their participation in cholesterol uptake and trafficking, as well as their association with molecules involved in phagocytosis. We also provide evidence that cholesterol depletion and trafficking arrest as well as *Ehnpc1* and *Ehnpc2* genes knockdown, affect virulence, particularly erythrophagocytosis and cell motility.

## Results

### *E*. *histolytica* possesses one *npc1* (*Ehnpc1*) and two *npc2* (*Ehnpc2a*, *Ehnpc2b*) genes and their respective proteins bind cholesterol

**Fig 1:**
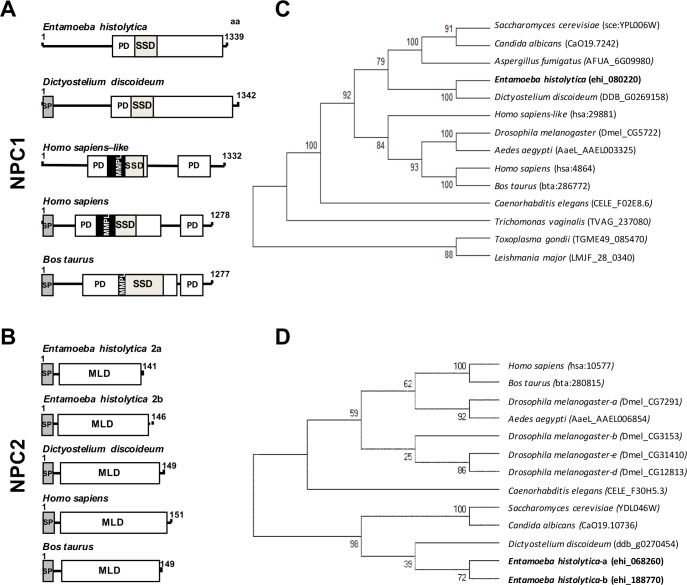
Structural domains and phylogenetic trees of EhNPC1, EhNPC2a and EhNPC2b. (A, B) Schemes show the main structural characteristics of NPC1 (A) and NPC2 (B) from distinct organisms. PD: patched domain, SSD: sterol sensing domain, MMPL: putative integral membrane domain, SP: signal peptide, MLD: MD-2 related lipid recognition domain. Numbers at the right correspond to the amino acids forming the proteins. (C, D) Phylogenetic tree indicating the position of *E*. *histolytica* NPC1 (C), NPC2a and NPC2b (D) proteins among different species. Numbers on horizontal lines in the trees indicate the confidence percentages of the tree topology from bootstrap analysis of 1000 replicates.

Our exploration in the AmoebaDB (http://amoebadb.org/amoeba/) revealed the presence of a 1339 amino acid sequence (EHI_080220) with 19.7 to 35% identity to NPC1 proteins of different species (Fig 1A, S1 Table). The putative EhNPC1 protein exhibited the patched (PD) and the SSD domains present in all reported NPC1 proteins [29]. However, it did not show the MMPL domain, found in bacteria as a putative integral membrane protein domain [30]. This domain is also absent in *Dictyostelium discoideum* NPC1 [31] (Fig 1A). We also found two sequences of 141 (EHI_068260) (EhNPC2a) and 146 (EHI_188770) (EhNPC2b) amino acids containing the MLD domain, which is a NPC2 signature [15] (Fig 1B). These sequences showed 29.4% identity between them and 11 to 26% with other NPC2 proteins (S2 Table).

The full length amino acid sequences of EhNPC1, EhNPC2a and EhNPC2b were compared with NPC1 and NPC2 protein sequences from other organisms to construct phylogenetic trees using the MEGA 5.05 software. EhNPC1 was grouped in a single clade with *D*. *discoideum* NPC1 in the branch of slime molds, yeasts and filamentous fungus and far from hypothetical NPC1s from protozoa such as *Trichomonas vaginalis*, *Toxoplasma gondii* and *Leishmania major* (Fig 1C). Interestingly, EhNPC2a and EhNPC2b also displayed a close relationship with *D*. *discoideum* NPC2 in the branch of slime molds and yeasts (Fig 1D).

**Fig 2:**
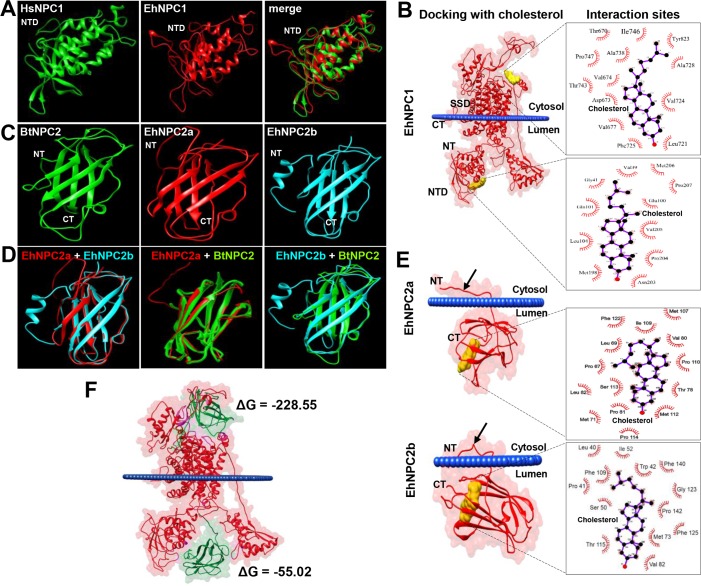
3D structures of NTD-EhNPC1, EhNPC2a and EhNPC2b and molecular dockings between them and with cholesterol. (A) NTD-EhNPC1 model (1 to 250 amino acids) predicted by RaptorX server was compared with the crystal of human NTD-NPC1 protein (HsNPC1) (23 to 254 amino acids). (B) Docking simulation of EhNPC1 with cholesterol performed using the AutoDock Tools V1.5.6 program. SSD: sterol sensing domain. NTD: amino terminal domain. (C, D) The EhNPC2a and EhNPC2b 3D structures (full-length amino acid sequences) were compared between them and with the NPC2 *B*. *taurus* crystal (BtNPC2). (E) Docking simulation of EhNPC2a and EhNPC2b with cholesterol. Arrows: amino acids tail at the amino terminus that it is not present in the crystal of *Btaurus* NPC2. Squares at the right in B and E show the amino acids involved in the protein-cholesterol interaction. (F) Docking simulation of EhNPC1 and EhNPC2a binding. ΔG: binding energy. Blue lines in dockings: plasmatic membrane. NT: amino terminus. CT: carboxy terminus.

To obtain further evidence on the structural relationship of EhNCP1, EhNPC2a and EhNPC2b with their respective orthologues, we constructed their 3D models using RaptorX server (http://raptorx.uchicago.edu). The predicted 3D structure of NTD EhNPC1 (1–248 amino acids) presented 92% structural identity to the *Homo sapiens* NTD NPC1 crystal (23 to 252 amino acids) [20]; and their merged images extensively overlapped (Fig 2A). EhNPC1 appeared as a membrane protein with 16 transmembrane domains, two structural regions located in the endosomal lumen, where the NTD is found, as described for other organisms [20], and a single larger domain in the cytosolic side (Fig 2B).

Docking analysis revealed two main interaction sites of EhNPC1 with cholesterol. According to *in silico* predictions, one site was located in the cytosol and the other at the NTD, in the endosomal lumen (Fig 2B). Results evidenced hydrophobic interactions of cholesterol with 13 amino acids of EhNPC1 at cytoplasmic domain (Thr670, Asp673, Val671, Val677, Leu721, Val724, Phe725, Ala728, Ala738, Thr743, Ile746, Pro747 and Tyr823); and with eleven residues of the NTD (Val39, Gly41, Glu100, Gln101, Leu104, Met198, Asn203, Pro204, Val205, Met206 and Pro207) (Fig 2B). The binding energies for these sites were ΔG ═ -8.6 and ΔG ═ -7.3 Kcal/mol, respectively, indicating a weak binding.

The 3D structures of EhNPC2a and EhNPC2b proteins presented 83.2% structural identity between themselves, and 88.6 and 86% with the *Bos taurus* crystal [32], respectively. EhNPC2 proteins exhibited seven beta strands (Fig 2C and 2D), as described for other NPC2 [32]. Interestingly, EhNPC2a and EhNPC2b displayed an extra amino acid tail at the N-terminus formed by 14 and 18 residues, respectively (Fig 2C–2E) that have not been reported in other NPC2 orthologues. The Orientation of Proteins in Membranes (OPM) database (http://opm.phar.umich.edu/) predicted that in EhNPC2a, Leu11, Phe12, Ala13 and Ala14 residues were attached to the membranes (Fig 2E); whereas in EhNPC2b the residues in contact with membranes were Thr19, Ala18, Leu17, Met16, Leu12 and Phe6 (Fig 2E), these results suggest that both EhNPC2 could be peripheral membrane proteins, which allow them to cross the membranes during cholesterol transport. The EhNPC2a interaction with cholesterol was predicted to be carried out by hydrophobic bonds corresponding to Pro67, Leu69, Met71, Thr78, Val80, Pro81, Leu82, Met107, Ile109, Pro110, Met112, Ser113 and Phe122 residues (Fig 2E). By contrast, EhNPC2b binds to cholesterol through Leu40, Pro41, Trp42, Ser50, Ile52, Met73, Val82, Thr115, Gly123, Phe125, Phe140 and Pro142 residues (Fig 2E). Amino acids in bold are in comparable positions and separated by the same number of residues in both proteins. The cholesterol binding energies to EhNPC1 and EhNPC2 were also low: ΔG ═ -9 and -10.15 Kcal/mol, respectively.

We also performed docking analysis using EhNPC1 and EhNPC2. Interestingly, results showed that EhNPC1 presents two putative interaction sites with EhNPC2, in cytosol and endosomal lumen (Fig 2F), with ΔG ═ -228.5 and -55.02 Kcal/mol, respectively. ΔG values reflect a stronger binding between the proteins compared to those with cholesterol. However, these values are still comparably low, corresponding to rather short-lived interactions. In summary, our bioinformatics analysis revealed that EhNPC1, EhNPC2a and EhNPC2b are NPC1 and NPC2 orthologues [21] that interact between them and with cholesterol, suggesting that they could play an important role in cholesterol transport.

### *Ehnpc1*, *Ehnpc2a* and *Ehnpc2b* genes are expressed in trophozoites

**Fig 3:**
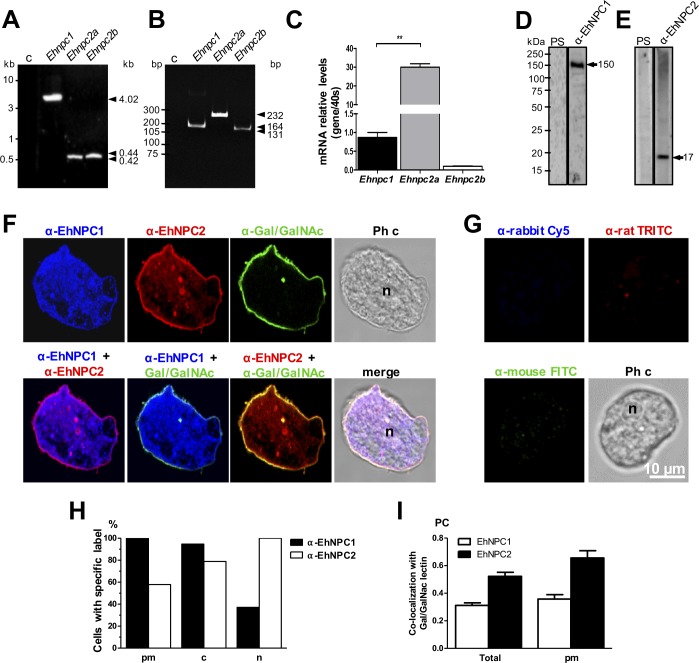
Expression and localization of EhNPC1 and EhNPC2 in trophozoites. (A) *Ehnpc1*, *Ehnpc2a* and *Ehnpc2b* full-length genes were PCR amplified using specific primers and genomic DNA. (B) RT-PCR amplification of transcript fragments using specific primers and cDNA. c: Controls without gDNA or with mRNA as template. (C) The relative expression of the three genes was measured by RT-qPCR in trophozoites, using as a control the *40s ribosomal* S2 protein gene. ** p<0.01. (D, E) Total extracts of *E*. *histolytica* were separated by 10% SDS-PAGE and analyzed by western blot assays using pre-immune serum (PS) or rabbit α-EhNPC1 (D) or rat α-EhNPC2 (E) antibodies. (F) Representative images of laser confocal microscopy of PFA-fixed trophozoites using rabbit α-EhNPC1 or rat α-EhNPC2 or mouse α-Gal/GalNAc lectin antibodies. (G) Controls using only secondary antibodies. Ph c: phase contrast images. (H) Protein localization of EhNPC1 and EhNPC2 in plasma membrane (pm), cytoplasm (c) or nucleus (n). Counts were performed in 50 cells. (I) Pearson coefficient (PC) correlation measured in at least 15 confocal images, indicating co-localization of EhNPC1 or EhNPC2 with Gal/GalNAc lectin in the entire cell and in the plasma membrane. Laser sections = 0.5 μm.

To confirm that the genes found *in silico* were *bona fide* genes in the parasite, we designed specific primers (S3 Table) using sequences obtained from the AmoebaDB. PCR amplification from genomic DNA gave 4.0, 0.42 and 0.44 kbp fragments, corresponding to the expected size of *Ehnpc1*, *Ehnpc2a* and *Ehnpc2b* full length genes, respectively (Fig 3A). Sequencing of the three genes revealed full open reading frames, no introns and 100% identity to the genes annotated in the AmoebaDB. RT-PCR assays evidenced bands at the expected size, indicating that genes are transcribed (Fig 3B). Intriguingly, RT-qPCR assays showed that *Ehnpc2a* was expressed 30 folds more than *Ehnpc1* gene, whereas, *Ehnpc2b* was poorly expressed (Fig 3C).

We generated an antibody against an EhNPC1 specific polypeptide (766-DEQPMYDKDGQYVPVEKRLE-785) that detected in western blot assays the expected single 150 kDa band in trophozoites samples (Fig 3D). Antibodies against EhNPC2a were produced in rats using recombinant proteins. They revealed the expected 17 kDa band (Fig 3E) and it was used as a pan antibody because they recognized both EhNPC2 recombinant proteins (S1 Fig), furthermore, the *Ehnpc2a* gene is transcribed more efficiently than *Ehnpc2b*. Pre-immune sera did not recognize any band (Fig 3D and 3E).

Confocal images showed that in basal conditions (trophozoites cultured in TYI-S medium, without stimulus), 100 and 60% of trophozoites were labeled in plasma membrane by α-EhNPC1 and α-EhNPC2 antibodies, respectively (Fig 3F and 3H), co-localizing with the α-Gal/GalNAc lectin antibody, used as a plasma membrane marker (Fig 3F). However, Pearson coefficient (PC) showed a higher co-localization of Gal/GalNAc lectin with EhNPC2 (Fig 3I). 100 and 80% of trophozoites showed α-EhNPC1 and α-EhNPC2 antibodies label in the cytoplasm, and only 40 and 100% of trophozoites, respectively, presented fluorescence in the nuclei (Fig 3F and 3H). We do not know yet the function, if any, of EhNPC1 and EhNPC2 in the nucleus. In all experiments reported here, we used as controls only the secondary antibodies, which always gave negative results (Fig 3G).

**Fig 4:**
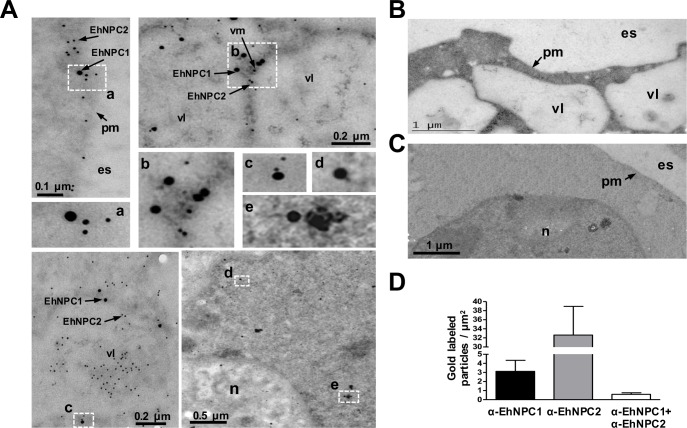
Localization of EhNPC1 and EhNPC2 in trophozoites analyzed by TEM. (A) Thin sections of trophozoites were incubated with rabbit α-EhNPC1 and rat α-EhNPC2 antibodies, followed by incubation with gold labeled α-rabbit and α-rat secondary antibodies (20 and 10 nm gold particles, respectively). Squares indicate the magnified areas marked with the corresponding lower case letters. pm: plasma membrane, vl: vesicle lumen, vm: vesicle membrane, es: extracellular space, n: nucleus. (B, C) Controls using only secondary antibodies. (D) Graph showing number of EhNPC1 and EhNPC2 molecules recognized by the respective gold-labeled antibodies and their co-localization.

TEM gold immunolabeling assays confirmed the polytopic cellular location of these proteins (Fig 4A). Images evidenced that in basal conditions, the majority of EhNPC1 and EhNPC2 proteins appeared separated, some of them localized close each other and only few appeared together (Fig 4A–4D). EhNPC1 and EhNPC2 were localized at plasma membrane, in the vesicle lumen and membranes. The labeled vesicles could correspond to endosomes; in there EhNPC1 was frequently surrounding them and EhNPC2 appeared inside (Fig 4A). Controls using only the secondary antibodies gave none or scarce signals (Fig 4B and 4C). These results together evidenced that *Ehnpc1* and *Ehnpc2* genes are transcribed and translated and their proteins are localized in several cellular regions, indicating that they are highly mobile proteins, which is in agreement with their hypothetical function as cholesterol transporters.

### After serum stimulus, EhNPC1 and EhNPC2 co-localize with cholesterol

**Fig 5:**
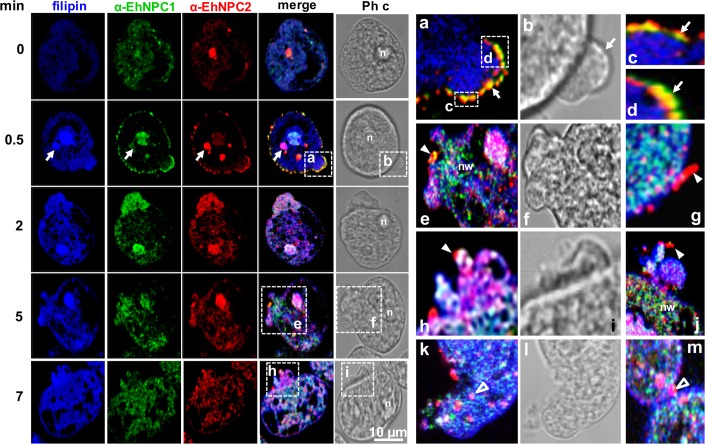
Localization of EhNPC1 and EhNPC2 in trophozoites after an ABS pulse. Trophozoites were serum starved for 12 h by culturing in TYI medium on coverslips. Then, ABS was added as a cholesterol source at 37°C for 0.5 to 7 min. Cells were washed, fixed and incubated with filipin, rabbit α-EhNPC1 and rat α-EhNPC2 antibodies, secondary antibodies, and examined by laser confocal microscopy. Arrows: cytoplasmic dots. Squares marked with lower case letters are magnified at the right. (a-d) Membrane vacuoles containing EhNPC1 and EhNPC2. Arrows: show the plasma membrane with EhNPC2 or EhNPC1 facing the extracellular space. (e) Networks stained by α-EhNPC1 antibody. (e-j) rod and spherical structures (arrowheads) facing the extracellular space, stained mainly by α-EhNPC2 antibody. (k-m) Spherical structures of 1 to 2 μm (empty arrowheads) inside the cell. n: nucleus, Ph c: phase contrast images, nw: networks like structures.

The main source of cholesterol in trophozoites in culture comes from adult bovine serum (ABS). We explored the participation of EhNPC1 and EhNPC2 in cholesterol trafficking after a serum stimulus. After serum starving of trophozoites, confocal images revealed that both proteins were barely present in the plasma membrane and cholesterol was faintly stained by filipin, a highly specific compound for cholesterol detection (Fig 5). Immediately after ABS addition (0.5 min), filipin detected cholesterol in the cytoplasm and nucleus, together with EhNPC1 and EhNPC2 (Fig 5). At this time, EhNPC1 and EhNPC2 co-localized in vesicles and in a uniform punctuate pattern at the plasma membrane (Fig 5A–5D). In most cases a yellow area was evident in membrane protrusions, indicating co-localization of both proteins (Fig 5A–5D). We also observed both proteins out of the cell (Fig 5G and 5J). After 2 to 5 min, membrane protrusions or putative vesicles appeared carrying cholesterol, and, in most of them, EhNPC2 was outwardly (Fig 5E and 5K), strengthening the hypothesis on the mobilization of both proteins during cholesterol capture. Number and size of membrane protrusions or putative vesicles increased through incubation time, and both proteins were close to cholesterol-containing vesicles outside and inside of the cell (Fig 5A, 5B, 5E, 5F, 5H and 5I). In some trophozoites, membrane protrusions or putative vesicles appeared at a cellular pole, in a “cap” model (Fig 5, 5 min), but they were also seen in other parts of the plasma membrane (Fig 5, 7 min). Outside of the cell, joined or not to the plasma membrane, EhNPC2 was revealed in vacuoles with rod and sphere shaped forms (Fig 5G–5M). Network-like structures mainly formed by EhNPC1 and cholesterol were observed inside the cells, adjacent to the networks formed mainly by EhNPC1 and cholesterol, and inside EhNPC2-containing spheres (1.5 to 2 μm) also appeared there (Fig 5E–5J). The spherical structures may correspond to the vesicles detected by TEM (Fig 4). These findings strongly suggest that cholesterol promotes the mobilization and secretion of EhNPC1 and EhNPC2.

**Fig 6:**
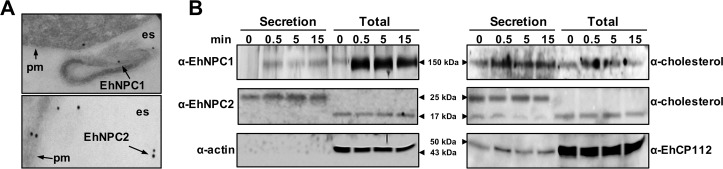
Secretion of EhNPC1 and EhNPC2 after ABS stimulus. (A) TEM of trophozoites under basal condition showing EhNPC1 and EhNPC2 in the extracellular space. pm: plasma membrane, es: extracellular space. (B) After the ABS stimulus, supernatants were collected and trophozoites were lysed at the indicated times. Secreted products and total extracts were analyzed by SDS-PAGE and western blot assays using rabbit α-EhNPC1, rat α-EhNPC2, rabbit α-cholesterol, rabbit α-EhCP112 and mouse α-actin antibodies and corresponding secondary antibodies. Actin was used as a control of cell integrity and EhCP112 as a secretion control.

In TEM images, we detected EhNPC1 and EhNPC2 in the extracellular space of trophozoites in basal conditions (Fig 6A). To explore whether the proteins were secreted, we performed secretion assays, giving an ABS pulse for different times to serum-starved trophozoites. In agreement with the confocal microscopy images (Fig 5, 0 min), EhNPC1 and EhNPC2 were poorly detected in secreted products of starved trophozoites. However, they were found in the supernatants of trophozoites challenged with ABS for 0.5 min (Fig 6B), indicating that they were secreted in response to the ABS pulse. Surprisingly, the α-EhNPC2 antibody did not reveal the expected 17 kDa band, but only a 25 kDa band was detected in the secreted products. This aberrant migration could be due to the binding of EhNPC2 to cholesterol present in the serum. To get further evidence on this, the nitrocellulose membranes were re-blotted using an α-cholesterol antibody. This antibody detected a band with similar migration than EhNPC1 and two bands of 17 and 25 kDa, corresponding to those detected by the α-EhNPC2 antibody. EhCP112, used as a positive secretion control, was found in both, supernatant and trophozoites extracts at all times, whereas actin, used as a negative control for secretion, was only detected in trophozoites samples. These results strongly suggest that these proteins are associated with cholesterol. A hypothesis could be that vesicles containing EhNPC1 and EhNPC2 come from and back to the cell, possibly carrying cholesterol from outside.

### EhNPC1 and EhNPC2 co-localize with cholesterol during phagocytosis

**Fig 7:**
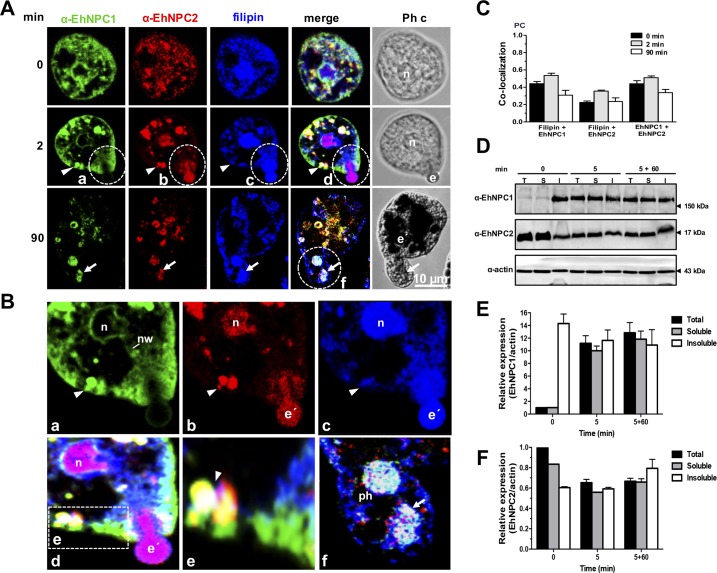
Localization of EhNPC1, EhNPC2 and cholesterol during phagocytosis. (A) Trophozoites were incubated with erythrocytes (1:25) at 37°C, for the indicated times. Then, samples were prepared for laser confocal microscopy and stained with filipin, rabbit α-EhNPC1 and rat α-EhNPC2 antibodies and secondary antibodies. Ph c: phase contrast images, e’: erythrocytes, n: nucleus. Arrow head: cytoplasmic vesicles. (B) (a-d) Dotted circles: Magnification of an erythrocyte that is being internalized. (e) Magnification of vesicles close to the plasma membrane. (f) Magnification of a huge phagosome (ph) containing digested erythrocytes (arrows). nw: networks. (C) PC of the co-localization between filipin and EhNPC1 or EhNPC2 or EhNPC1 with EhNPC2 at distinct phagocytosis time. (D) Trophozoites were incubated with erythrocytes at 37°C for 5 min, then adherent and non-ingested erythrocytes were removed by washing with a mixture of water-TYI medium. Later, trophozoites were incubated at 37°C again for 60 min with TYI to continue the process. Trophozoites were lysed and also cellular fractionation was carried out. Samples were processed for 12% SDS-PAGE and western blot assays. Representative blots of three experiments are shown. T: total extracts, S: soluble fraction, I: insoluble fraction. (E,F) Densitometry of the bands in (D) corresponding to EhNPC1 (E) and EhNPC2 (F) using actin as loading control.

As phagocytosis is one of the main mechanisms for nutrients uptake and virulence expression in the parasite and trophozoites have an intense membrane synthesis during the event, we analyzed the EhNPC1 and EhNPC2 behavior when cells ingest erythrocytes. Fig 7 shows images of the most relevant facts observed in different experiments. Under basal conditions, both proteins and cholesterol were found at the plasma membrane, cytoplasm, and nuclei (Fig 7A, 0 min). Immediately after sensing the presence of erythrocytes, EhNPC1 and EhNPC2 proteins moved to the contact sites at the plasma membrane (Fig 7A and 7B); and they were present in cytoplasmic vesicles with cholesterol inside (Fig 7, 2 min). Interestingly, EhNPC2 co-localized with cholesterol of phagocytosed erythrocytes (Fig 7A and 7B). Both proteins were observed in trophozoites as a continuous dot pattern from the partially ingested erythrocytes to the cytoplasm (Fig 7A, 2 min; 7Ba-d). EhNPC1 was seen in the external part of the phagocytic cup (Fig 7A and 7B). Between the nucleus and erythrocytes, networks-like structures were observed decorated mainly by the α-EhNPC1 antibody and filipin, and spots recognized by α-EhNPC2 antibodies (Fig 7A, 2 min; 7B). At 90 min, cholesterol appeared distributed at the plasma membrane, and in vacuoles inside other huge vacuoles that may correspond to phagolysosomes and MVBs containing digested erythrocytes, and co-localizing with EhNPC2 and EhNPC1 (Fig 7A, 90 min; 7Bf). Co-localization of both proteins was not higher than 0.59 (Fig 7C), suggesting that they do not interact all the time. Recently, we have identified LBPA and EhADH inside phagolysosomes and MVBs after 60 and 90 min of phagocytosis [26]. By their size and appearance at late times of phagocytosis, we presume that they correspond to the ones detected here containing cholesterol, EhNPC1 and EhNPC2.

Then, we performed erythrophagocytosis assays in a pulse-chase model to detect protein mobilization by western blot assays. In these experiments, trophozoites were incubated with erythrocytes for 5 min, then, free and adhered erythrocytes were removed by washing samples; and the phagocytic process of the already ingested erythrocytes continued at 37°C for different times. Total proteins and the soluble and insoluble fractions of lysed trophozoites taken at 0, 5 and 5+60 min of phagocytosis were identified by western blot assays. In basal conditions (0 min), EhNPC1 was detected enriched in the insoluble fraction. However, after 5 and 5+60 min of phagocytosis the protein was similarly distributed in the three samples (Fig 7D). By contrast, under basal conditions, EhNPC2 was more abundant in the soluble fraction (Fig 7D). Densitometry analysis using α-actin antibodies as loading control, confirmed this (Fig 7E and 7F). These findings suggest that in basal conditions EhNPC1 is more abundant in membranes, whereas EhNPC2 is concentrated in the cytoplasm; while during phagocytosis they are more homogenously distributed in the entire cells, evidencing the dynamic cholesterol trafficking.

### EhNPC1 and EhNPC2 are located in the ER

**Fig 8:**
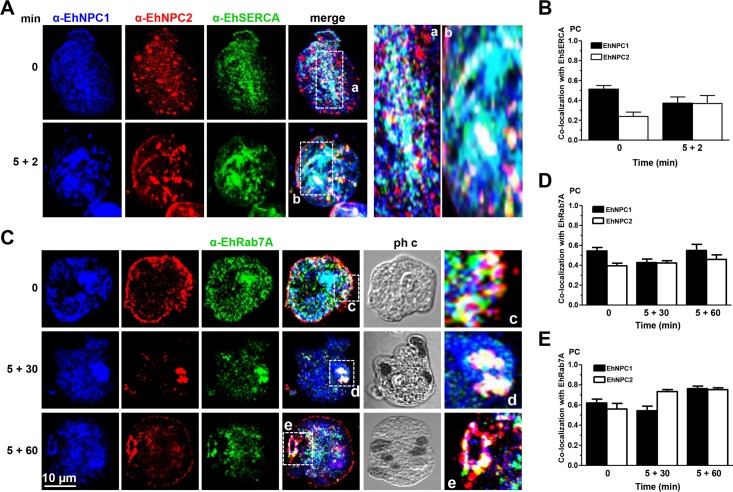
Co-localization of EhNPC1 and EhNPC2 with EhSERCA and EhRab7A. Trophozoites were incubated with erythrocytes at 37°C and treated as Fig 7. Samples were processed for confocal microscopy using α-EhNPC1, α-EhNPC2 and (A) α-EhSERCA or (C) α-EhRab7A antibodies. (a-e) Magnification of the white squares. (B, D, E) PC of the co-localization between EhNPC1 or EhNPC2 with EhSERCA (B), or with EhRab7A in the whole cell (D) or in cellular structures stained by the three antibodies (E).

In mammalian cells, NPC1 and NPC2 are located in the ER from where they distribute cholesterol to different organelles. Here we searched for EhNPC1 and EhNPC2 in the ER. Confocal images of trophozoites in basal conditions showed that both proteins co-localized in networks and vacuoles with EhSERCA, an ER marker [33] (Fig 8Aa,b). EhNPC2 also appeared as 1.5 to 2 μm spheres in the cytoplasm (Fig 8A). After 5+2 min phagocytosis, co-localization did not vary significantly (Fig 8A), suggesting that EhNPC1 and EhNPC2 proteins maintain a constant presence in ER, as it has been described for other eukaryotes [34]. PC values of co-localization of EhNPC1 and EhNPC2 with EhSERCA were between 0.25 and 0.51 (Fig 8B). These findings corroborate that the proteins detected around the nuclei are indeed in the ER.

### EhNPC1 and EhNPC2 co-localize with EhRab7A in endosomes.

The Nozaki group has found that EhRab7A is involved in the retrograde transport from phagosomes to the Golgi apparatus and it is located mainly in late endosomes [35, 36]. Based on their elegant work, we explored whether spherical structures containing EhNPC2 detected in trophozoites, corresponded to endosomes. By laser confocal microscopy, the α-EhRab7A antibody recognized these cytoplasmic structures, which increased in size throughout the chase-time phagocytosis assays (Fig 8C), evidencing that they are endosomes. In basal conditions and throughout phagocytosis, EhRab7A was associated with EhNPC1 and EhNPC2; frequently forming donut-like structures that may be phagolysosomes and MVBs (Fig 8Cc-e). PC values for EhNPC1 and EhNPC2 with EhRab7A were 0.39 to 0.55 in the entire cell (Fig 8D) and increased to 0.47 to 0.78 in the donut-like structures (Fig 8E). These results strengthen the assumption that EhNPC1 and EhNPC2 are also in endosomes.

### EhNPC1 and EhNPC2 associate with endosomal molecules in acidic vesicles

**Fig 9:**
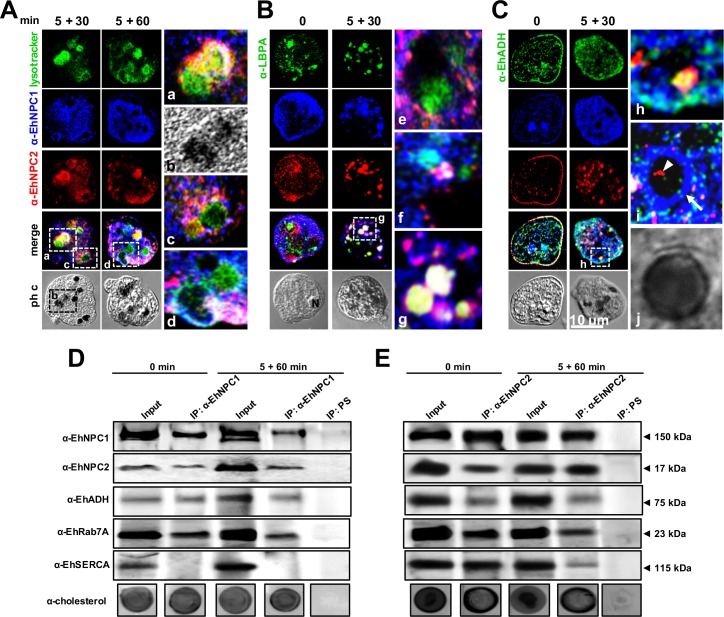
Co-localization and association of EhNPC1 and EhNPC2 with cholesterol and endosomal molecules. Trophozoites were incubated with erythrocytes as in Fig 7 and processed for confocal microscopy after incubation with α-EhNPC1 and α-EhNPC2 antibodies and (A) Lysotracker or (B) α-LBPA or (C) α-EhADH antibody and corresponding secondary antibodies. (a-d,g,h) Magnification of white squares. (e,f,i,j) Magnifications from other images. Ph c: phase contrast images, arrow in I: EhNPC1 outside endosomes, arrowhead: EhNPC2 inside endosomes. D, E) Trophozoites in basal condition or after 5+60 min of erythrophagocytosis were lysed and immunoprecipitation assays (IP) were performed using α-EhNPC1 (D) or α-EhNPC2 (E) antibodies or preimmune serum (PS). Immunoprecipitated proteins of trophozoites were analyzed by western blot and dot blot experiments, using α-EhNPC1, α-EhNPC2, α-EhADH, α-EhRab7A, α-EhSERCA and α-cholesterol antibodies.

In mammals, NPC1 binds to cholesterol in late endosomes in an acidic milieu where LBPA and Alix protein participate in cholesterol homeostasis [22]. We hypothesized that cholesterol containing vesicles inside phagolysosomes, together with EhNPC1 and EhNPC2, also contain LBPA and EhADH [25, 26]. Thus, we investigated the nature of these vesicles during phagocytosis using Lysotracker, a marker of acidic vesicles. After 5+30 and 5+60 min of erythrophagocytosis, more than 90% of the phagosomes were positive for α-EhNPC1, α-EhNPC2 antibodies and Lysotracker (Fig 9A). Lysotracker appeared in phagolysosomes and MVBs, containing partially digested erythrocytes (Fig 9Aa-d). These results show that EhNPC1 and EhNPC2 are in an acidic milieu characteristic of phagolysosomes.

Besides, at 5+30 min, all phagolysosomes showed EhNPC1, EhNPC2 and LBPA (Fig 9Be-g). Moreover, EhADH co-localized with EhNPC1 or EhNPC2 under basal conditions, mainly in the plasma membrane, and, during phagocytosis, in phagolysosomes and MVBs (Fig 9Ch-j). Immunoprecipitation assays using α-EhNPC1 antibody confirmed that this protein is associated under basal conditions and during phagocytosis with EhNPC2, EhADH and EhRab7A, but not with EhSERCA (Fig 9D), However, α-EhNPC2 antibody precipitated all of these proteins including EhSERCA, suggesting that at some point these proteins are interacting (Fig 9E). Cholesterol was also detected in dot blots in both immunoprecipitates (Fig 9D and 9E). These results evidenced that cholesterol, LBPA, EhADH, EhRab7A, EhNPC1 and EhNPC2 co-localize in phagolysosomes and MVBs, probably cooperating to facilitate the phagocytosis-digestion processes; and our data also reinforce the association of EhNPC1 and EhNPC2 with cholesterol.

### Trophozoites with stocked cholesterol diminish their rate of phagocytosis and do not disturb the intestinal barrier

**Fig 10:**
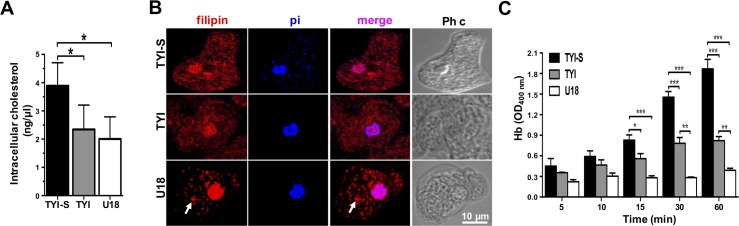
Erythrophagocytosis of trophozoites cultured in TYI-S, TYI and TYI plus U18. Trophozoites were incubated ON at 37°C in TYI-S, TYI and TYI plus U18. (A) Cholesterol concentration in the respective trophozoites was measured as described in material and methods. (B) Laser confocal microscopy showing the morphology and the cholesterol localization by filipin staining. Nuclei were counterstained with propidium iodide (pi). Images were false colored to obtain a better contrast. Ph c: phase contrast images, arrows: cytoplasmic dots. (C) Rate of erythrophagocytosis spectrophotometrically measured by hemoglobin (Hb) concentration inside trophozoites.

To evaluate the effect of cholesterol trafficking on phagocytosis, we used the U18666A (3-β-[(2-diethyl-amino)ethoxy]androst-5-en-17-one) (U18) drug that binds to NPC1 in the SSD, blocking the cholesterol traffic [37]. For these experiments, trophozoites cultured without serum (TYI), to eliminated part of the cholesterol source, showed less than 50% of the cholesterol concentration that the cells cultured in basal conditions (with serum). Trophozoites cultured without serum and in the presence of U18 presented slight differences in cholesterol concentration (Fig 10A). By confocal microscopy, U18 treated trophozoites, showed filipin-positive abundant clumps in the cytoplasm suggesting cholesterol accumulation (Fig 10B). These results suggest that in *E*. *histolytica*, U18 also interferes with the cholesterol trafficking, but not with the cholesterol amount inside the trophozoites (Fig 10A and 10B). However, U18 treated cells ingested at 60 min, 2 erythrocytes/trophozoite, whereas trophozoites cultured in TYI medium engulfed about 6 erythrocytes, and trophozoites cultured in TYI-S medium ingested a mean of 18 erythrocytes (Fig 10C). These findings suggested that the cholesterol arresting by U18 interferes with the rate of phagocytosis.

**Fig 11:**
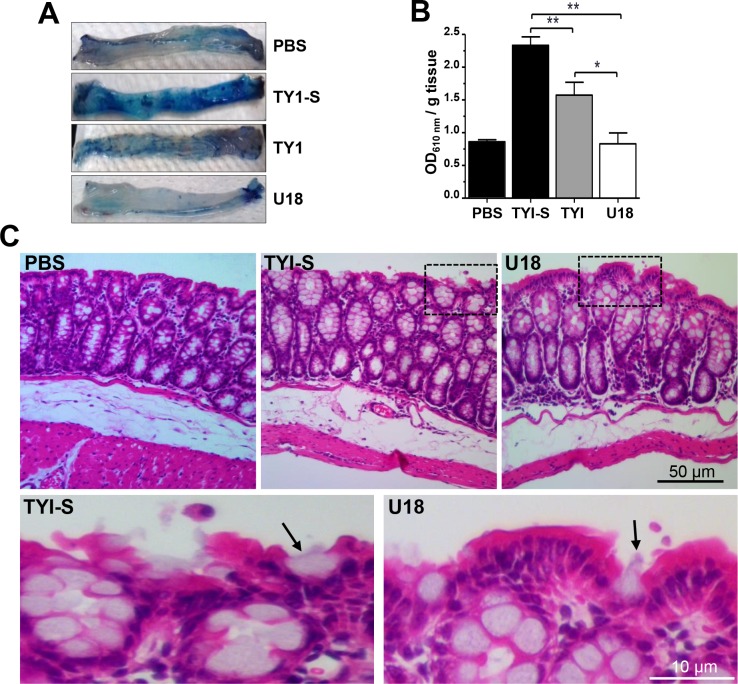
*In vivo* virulence of trophozoites cultured in TYI-S, TYI and TYI plus U18. Capability of trophozoites to impair the mouse intestinal barrier. (A) Distal parts of the mouse colons after treatment with trophozoites or with PBS. Intestinal barrier impairment was measured as the ability of the Evans blue dye to permeate the mouse intestinal epithelium after contact with trophozoites. n = 5. (B) Data represent the mean ± standard error. PBS: mice undergoing surgery, but not inoculated with trophozoites. * p<0.05, ** p<0.01. (C) Hematoxylin-eosin staining of tissues. Squares were magnified in the corresponding lower panels. Arrows: trophozoites.

We also analyzed the effect of the cholesterol trafficking arrest by using U18 in the amoebiasis intestinal mice model (strain C57BL/6) [38]. In this model, virulent trophozoites compromise the intestinal epithelial barrier, which could be monitored by Evans blue dye permeability [39]. The intestinal epithelium of mice inoculated with U18-treated trophozoites did not suffer damage, giving similar results to the negative controls (mice inoculated only with PBS), and showing that the epithelial barrier was not disrupted (Fig 11A and 11B). By contrast, trophozoites cultured in TYI induced 62% permeability in colonic epithelium compared to trophozoites cultured in TYI-S medium, that was taken as 100% (Fig 11A and 11B). Thus, while cholesterol depletion only ameliorated the capacity of trophozoites to induce intestinal epithelial permeability, U18 treatment completely inhibited it. Tissue sections of colon epithelium stained with hematoxylin-eosin showed that trophozoites grown in TYI-S medium provoked discontinuity in epithelial layer, cells rounding, and a higher number of cells layers, cellular infiltration and swelling, whereas animals inoculated with U18 treated trophozoites presented normal epithelium, similar to the negative controls (Fig 11C). Our results showed that cholesterol is necessary for trophozoites to ingest red blood cells, and to impair the intestinal epithelial barrier.

### Knockdown of *Ehnpc1* and *Ehnpc2* genes diminishes rate of phagocytosis and cell motility

**Fig 12:**
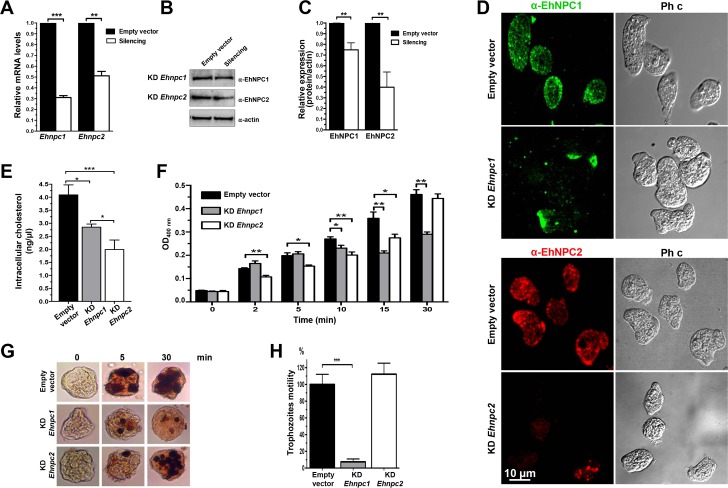
Silencing of *Ehnpc1* and *Ehnpc2* genes in *E*. *histolytica*. (A) Trophozoites clone G3 were transfected with *psAP-2*, *psAP-2Ehnpc1* or *psAP-2Ehnpc2a* plasmids and stable populations were selected with 4 μg/ml G-418. RT-qPCR assays were performed using mRNA from transfected trophozoites (empty vector, KD *Ehnpc1* and KD *Ehnpc2*), using specific primers for *Ehnpc1* and *Ehnpc2a* genes and as a housekeeping the *40s ribosomal* S2 protein gene. (B) Western blot assays of transfected trophozoites extracts, using α-EhNPC1 and α-EhNPC2 antibodies and respectively secondary antibody. As a loading control, the same membrane was reblotted with α-actin antibodies. (C) Densitometry analysis of bands showed in (B) normalized again actin protein. (D) Confocal microscopy of transfected trophozoites in basal conditions using α-EhNPC1 or α-EhNPC2 antibodies. Ph c: phase contrast. (E) Cholesterol concentration in the transfected trophozoites was measured as described in material and methods. (F) Rate of erythrophagocytosis of transfected trophozoites. * p<0.05, ** p<0.01. (G) Diaminobenzidine-stained trophozoites that ingested erythrocytes for different times. (H) Motility assays of transfected trophozoites cultured in transwell inserts. *** p<0.001.

Phagocytosis and cell motility require cholesterol for membrane synthesis and fluidity. To get further evidence that EhNPC1 and EhNPC2 proteins are responsible for transporting the cholesterol for these events, we knocked down trophozoites in the *Ehnpc1* and *Ehnpc2* genes, using trophozoites of clone G3 and the psAP-2 vector to transfect them [40]. Both types of silenced trophozoites (KD *Ehnpc1* and KD *Ehnpc2*) expressed 30 and 50% of *Ehnpc1* and *Ehnpc2* mRNA, respectively, compared with the G3 trophozoites transfected only with the empty vector (Fig 12A). Protein expression was also diminished in silenced trophozoites in basal conditions; although, surprisingly, protein level decreased more in EhNPC2 than in EhNPC1 (Fig 12B and 12C), even when *E*. *histolytica* has two *Ehnpc2* genes that are transcribed (Fig 3), and only *Ehnpc2a* was silenced. These suggest mechanisms for a fine regulation in the level or in the half-life of the proteins that might be elucidated in the future. Confocal microscopy images confirmed the decrease of both proteins in mutant trophozoites in basal conditions; and showed that proteins were re-localized (Fig 12D). In KD *Ehnpc1* trophozoites, EhNPC1 protein was polarized in the plasma membrane, whereas, in KD *Ehnpc2* cells, EhNPC2 was scarcely found in plasma membrane and it was detected as few cytoplasmic dots (Fig 12D). KD *Ehnpc2* trophozoites presented a lesser amount of intracellular cholesterol (1.9 ng/μl) than KD *Ehnpc1* (2.8 ng/μl) whereas, the trophozoites transfected with the empty vector presented 4.0 ng/μl (Fig 12E), strongly suggesting the participation of these proteins in cellular cholesterol uptake.

The rate of erythrophagocytosis was also modified in silenced trophozoites. In early times of phagocytosis (2 to 5 min), KD *Ehnpc1* and control trophozoites ingested the same amount of erythrocytes (6 erythrocytes/trophozoite). However, after 5 min, KD *Ehnpc1* trophozoites arrived to a plateau, ingesting a mean of 10 erythrocytes/trophozoite, in comparison with control trophozoites that continued erythrocytes ingestion, reaching 18 erythrocytes/trophozoite at 30 min (Fig 12F and 12G). On the other hand, from 2 to 15 min of phagocytosis, the KD *Ehnpc2* trophozoites ingested a mean of 4 to 10 erythrocytes/trophozoite in comparison with control trophozoites, which ingested a mean of 5.6 to 14.4 erythrocytes/trophozoite. However, at 30 min of phagocytosis, KD *Ehnpc2* trophozoites recuperated the rate of phagocytosis and ingested a mean of 18 erythrocytes/trophozoite, similar to control trophozoites (Fig 12F and 12G). These results indicate that in the KD *Ehnpc2* trophozoites, the rate of erythrophagocytosis was affected only in early times, whereas, in KD *Ehnpc1* trophozoites, the erythrophagocytosis was affected after 10 min of contact with erythrocytes.

To evaluate the impact of EhNPC1 and EhNPC2 proteins on motility, we placed the transfected trophozoites in the upper chamber of transwell inserts and counted the number of trophozoites that were able to move toward the lower chamber, following a serum stimulus. Interestingly, after 3 h incubation, KD *Ehnpc1* trophozoites remained in the upper chamber, showing migration incapacity, whereas, KD *Ehnpc2* trophozoites showed similar motility than the control. (Fig 12H). These findings altogether revealed that EhNPC1 and EhNPC2 proteins have no redundant functions but they cooperate during cholesterol trafficking.

## Discussion

Using *in silico* analysis and distinct experimental approaches, we have shown here that the early emerging protozoan *E*. *histolytica* has one *Ehnpc1* and two *Ehnpc2* genes. In contrast, most eukaryotes have two *npc1* (*npc1* and *npc1l1*) and one *npc2* genes [14, 29, 32]. Molecular docking, co-localization and immunoprecipitation assays revealed that EhNPC1 and EhNPC2 proteins can interact between them and with cholesterol. Moreover, they associate with EhRab7A and EhADH proteins, whereas only EhNPC2 interacts with EhSERCA, although both proteins co-localized, immunoprecipitation assays showed no interaction between them at the times explored. Experiments with silenced trophozoites in *Ehnpc1* and *Ehnpc2* genes gave evidence that their corresponding proteins do not carry out redundant functions, but they cooperate in the cholesterol trafficking. The relevance of our findings lies in three main facts: firstly, cholesterol is fundamental for endocytosis and motility; both important for nutrition and virulence of *E*. *histolytica* [2–4, 8]. Secondly, cholesterol is not synthesized by trophozoites and very little is known on cholesterol trafficking in protozoa. In particular, the mechanism of cholesterol uptake and trafficking in *E*. *histolytica* trophozoites was almost unknown, except for the role of TMK39 protein in LDL proteins uptake [24], which could function in association with other proteins, such as EhNPC1 and EhNPC2, because it has not the canonical cholesterol binding sites [24]. In protozoa, except for a NPC1 like protein identified in *T*. *gondii* [41], no other references were found by us about the presence and function of NPC1 and NPC2; their genes only appear in the databases, but no experimental characterization has been done. This makes *E*. *histolytica* the first protozoa in which both genes and proteins has been identified and characterized. Thirdly, *E*. *histolytica* has a simple cholesterol trafficking, compared to other eukaryotes, because it depends on exogenous cholesterol, providing an excellent and less complicated model to elucidate this intricate event in eukaryotic cells.

NPC1 and NPC2 are ancient proteins, highly conserved throughout eukaryotic evolution [42]; to the extent that human and yeast *npc1* genes are genetically and functionally interchangeable [42]. Our results fortify this assumption by the high structural homology presented by human NTD-NPC1 and the *B*. *taurus* NPC2 crystals with EhNPC1 and EhNPC2 3D structures, respectively. The recent publication elucidating the 3D structure of the complete NPC1 protein in human [37] gives support to our model. In contrast to other eukaryotes, EhNPC2a and EhNPC2b present an amino acids tail with several residues that could be in contact with membranes. This, together with the positively charged protein regions [17], gives to EhNPC2 proteins the possibility of translocation across the plasma membrane and other organelle membranes, as our experimental approaches have suggested. These characteristics give further support to the hypothesis of the "hand-off" model, which can be working also in *E*. *histolytica* for cholesterol uptake and trafficking [19]. Immunoprecipitation assays using α-EhNPC1 or α-EhNPC2 antibodies confirmed that both proteins are associated, among them and with cholesterol, in trophozoites in basal conditions and during phagocytosis although sometimes they also appeared separated, supporting the "hand-off" model.

In this study we also asked: How do *E*. *histolytica* trophozoites bring exogenous cholesterol to their distinct organelles without a canonical LDL receptor?

Now, have evidences that EhNPC1 and EhNPC2 proteins carry cholesterol and participate in all processes from the sensing of external cholesterol sources to the digestion of the endocytosed products in phagolysosomes. Proximity and co-localization of EhNPC1 and EhNPC2 in the trophozoites surface immediately after ABS pulse and at the beginning of erythrophagocytosis, as well as their detection in secreted products, suggest that they are in charge of cholesterol capturing in *E*. *histolytica*. Our results do not discard the participation of other proteins, such as TMK39 protein.

**Fig 13:**
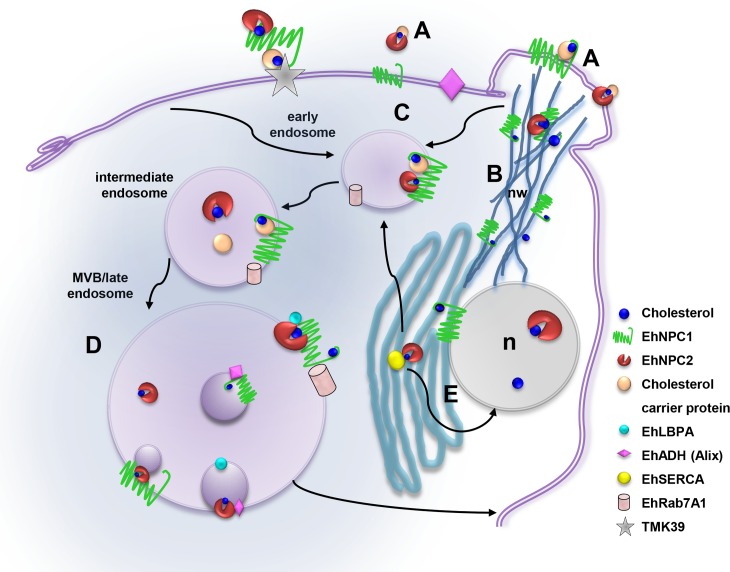
Working model of the EhNPC1 and EhNPC2 participation in cholesterol trafficking in *E*. *histolytica*. (A) Cholesterol uptake: EhNPC1 and EhNPC2 capture the cholesterol in the extracellular space and in the plasma membrane probably following the “hand-off” model (EhNPC1-cholesterol-EhNPC2-cholesterol-EhNPC1) and with the TMK39 participation. (B) Network-like structures formation: EhNPC1 and cholesterol form networks from cholesterol-containing membrane protrusions to ER, endosomes and nucleus that possibly facilitate cholesterol trafficking through EhNPC2 in a “hand-off” model. (C) Cholesterol influx: EhNPC1 and EhNPC2 associate with cholesterol and are internalized in EhRab-7A containing endosomes under basal conditions and during erythrophagocytosis. (D) EhNPC1 and EhNPC2 associate with phagolysosomal and MBVs molecules, particularly with LBPA and EhADH, which probably regulate cholesterol trafficking. (E) EhNPC1 and EhNPC2 accumulate in nucleus (n) and ER and may be distributed from there to other organelles.

Experiments using serum-starved trophozoites suggest that the external cholesterol is taken up into the cell by EhNPC1 and EhNPC2 in several steps: i) Immediately after sensing the cholesterol, EhNPC1 and EhNPC2 are organized in the plasma membrane in a punctuated pattern, co-localizing in some regions, but staying close and uncomplexed in some areas. It is plausible to speculate that EhNPC1 facilitates the transport of EhNPC2 to the plasma membrane to uptake exogenous cholesterol, but more experiments are necessary to probe this. ii) Next, small membrane projections or putative vacuoles that grow in size and amount throughout the endocytosis process appear at early times of endocytosis that are full of EhNPC1 and EhNPC2. iii) Then, EhNPC2-containing vesicles appear outside the cell, assuming rod- and sphere-like shapes that are in contact with vesicles full of cholesterol. It was also possible to distinguish in them small spots recognized by α-EhNPC1. Secretion assays confirmed that EhNPC1 and EhNPC2 are indeed released into the medium, probably to capture cholesterol. iv) EhNPC2 appears in vesicles co-localizing with cholesterol inside trophozoites, frequently making contact with smaller EhNPC1 containing vesicles. v) EhNPC1 and cholesterol formed networks from the plasma membrane to the nucleus and the ER; and also EhNPC2 appears there. vi) EhNPC1 and EhNPC2 accumulate in the ER and the nucleus. We do not know yet the significance of these proteins in the nucleus. vii) Cholesterol was driven to the plasma membrane and to vacuoles that are surrounded by EhNPC1 with EhNPC2 inside. All these steps are summarized in the cartoon of Fig 13.

Through erythrophagocytosis, most of these steps are also evident with some particularities. i) Co-localization of EhNPC1 and EhNPC2 is starting after two minutes contact or before. ii) Adhered, but not yet ingested, erythrocytes appear covered by EhNPC2 protein before target cell ingestion is finished. Trophozoites presented a channel, with partially ingested erythrocytes strongly stained by the α-EhNPC2 antibody and filipin, whereas the external part of the phagocytic cup was detected by α-EhNPC1 antibody. iii) At 30 and 90 min after phagocytosis, cholesterol appeared together with EhNPC1 and EhNPC2 in vacuoles, some with a relative uniform size, inside of phagolysosomes or MVBs. Interestingly, EhRab7A, LBPA, EhADH and cholesterol co-localized in late endosomes with EhNPC1 and EhNPC2, suggesting that they may participate in the cholesterol trafficking and homeostasis, as in mammalian cells [22]. vi) Cholesterol returned to plasma membrane following a similar movement than EhNPC1 and EhNPC2. It is possible that EhNPC1 and EhNPC2 tasks do not end until the digestion process is finished and no more cholesterol is available in the phagocytosed erythrocytes (Fig 13).

The phagocytosis process relies on cholesterol trafficking through the distinct type of endosomes formation. Here we used two strategies to evaluate this: i) we arrested the cholesterol trafficking by U18 drug which binds to the SSD sites in NPC1 [37, 43]. In our experiments we confirmed that the intracellular cholesterol concentration did not vary in comparison with TYI cultures trophozoites, but it accumulates in clumps and severely affected phagocytosis and *in vivo* virulence. Mice inoculated with U18 treated trophozoites did not present damage in the colon epithelium. ii) Experiments with trophozoites knocked down in *Ehnpc1* and *Ehnpc2a* genes gave strong support to the fact that both genes are important in cholesterol uptake. The diminish expression of proteins, provoked a reduction in the rate of phagocytosis. Interestingly, *Ehnpc1* knocked down trophozoites presented a dramatic decrease in motility, strongly suggesting a role of EhNPC1 protein in this function. We do not speculate on the role of EhNPC2 protein, because the presence of two genes and a possible compensatory mechanism in the cell could promote the transcription of the Ehnpc2b gene after the stimulus given by serum or erythrocytes. Another possibility is a half-life increase of the EhNPC2 protein. It is currently under study in our laboratory.

In conclusion, we provide the first evidence that *E*. *histolytica* presented two cholesterol transporting proteins, EhNPC1 and EhNPC2, that participate in exogenous cholesterol uptake and in its cellular trafficking, impacting in motility and phagocytosis. Future studies need to be addressed to fully elucidate remaining questions: How do EhNPC1 and EhNPC2 arrive to the plasma membrane and leave the cell? Combining our findings with results in other eukaryotes [44] we propose that EhNPC2 is transported by EhNPC1 to these places. How does EhNPC2 enter to the phagocytosed erythrocytes? We hypothesized that the “hand-off” model proposed for mammalian cells could be also applicable to *E*. *histolytica*. This implies that EhNPC2 moves to the plasma membrane, together with EhNPC1 and from there, cholesterol is “handed-off” from one protein to another, avoiding cholesterol trafficking through the hydrophobic medium, efficiently reaching its target organelles and facilitating the expression of virulence properties that require membrane synthesis and vacuoles fusion. The importance of cholesterol transport for the virulence and the involvement of EhNPC1 and EhNPC2 in this process, make these proteins promising targets for developing better strategies to defeat the amoebiasis.

## Materials and Methods

### *E*. *histolytica* cultures

Trophozoites of *E*. *histolytica*, clone A, strain HM1:IMSS [45] (already-existing collection) and clone G3 [40] were axenically grown at 37°C in TYI-S-33 medium (TYI-S) and harvested at logarithmic growth phase by chilling the culture flasks at 4°C [46]. For some experiments, trophozoites were cultured in TYI medium without serum during 12 h (TYI) or in TYI supplemented with 3 μg/ml of U18 during 12 h (Sigma-Aldrich). Then, fresh medium with or without the drug was added to proceed with the experiments. All experiments presented here were performed at least three times in duplicate.

### *In silico* analysis and phylogenetic trees construction

Human NPC1 (access number: hsa 4864) and bovine NPC2 (access number: bta 280815) protein sequences retrieved from the KEGG database (http://www.genome.jp/kegg/) were used as query to search putative *E*. *histolytica* EhNPC1 and EhNPC2 proteins and their orthologues. Structural domains were identified using the SMART genomics server (http://smart.embl-heidelberg.de/). Identity and e-value between EhNPC1 and EhNPC2 hypothetical proteins, compared with their orthologues, were determined using the Expert Protein Analysis System (ExPASy) of the Proteomic Analysis Server from the NCBI Blast service program (http://www.expasy.org/). The predicted amino acid sequences of EhNPC1, EhNPC2a and EhNPC2b (ehi_080220, ehi_068260 and ehi_188770, respectively) were aligned with orthologues sequences by ClustalW and data were submitted to phylogenetic analysis by UPGMA using MEGA 5.05 software [47]. Bootstrapping was performed for 1000 replicates.

### 3D structure modeling and molecular docking

The amino acid sequences of EhNPC1, EhNPC2a and EhNPC2b were analyzed using the RaptorX server (http://raptorx.uchicago.edu/) and predicted structures and orientation were obtained via the OPM database (http://opm.phar.umich.edu/). The 3D NTD EhNPC1 model (1 to 250 amino acids) was compared with the human NTD-NPC1 (3GKI) (23 to 254 amino acids) crystal, whereas the EhNPC2 3D structure (full amino acid sequence) was compared with the *B*. *taurus* NPC2 crystal (2HKA). Both crystals were retrieved from the Protein Data Bank (PDB). Templates of other NPC1 and NPC2 proteins, located at PDB, were used to obtain the EhNPC1, EhNPC2a and EhNPC2b 3D structures, which were visualized using the UCSF Chimera software. Molecular docking with cholesterol was performed for the three proteins using the AutoDockTools V1.5.6 program, and the interaction sites were analyzed by Ligplotv.4.5.3 program (http://www.ebi.ac.uk/thorntonsrv/software/LIGPLOT/). The grid size was 126x126x126 points with a 0.8 Å spacing. Dockings were performed with the empiric free energy function and the Lamarckian genetic algorithm. Number of GA runs was 200, using a maximum number for evaluation of 2x10^6^. Cholesterol structure was obtained from the ZINC12 database (zinc.docking.org) in PDB format and energy minimization was obtained through PRODGR (davapc1.boich.dundee.ac.us/prodrg/) and the CHIMERAV1.10.1 software.

### PCR and RT-PCR assays

Genomic DNA and total RNA were isolated from trophozoites using the Wizard Genomic DNA Purification kit (Promega) and Trizol reagent (Invitrogen), respectively, according to the manufacturer’s recommendations. cDNA was synthesized using oligo dT primers and the Superscript II reverse transcriptase (Invitrogen). PCR amplifications were carried out using 200 ng of DNA or cDNA as template and specific primers for *Ehnpc1 or Ehnpc2a* or *Ehnpc2b* genes (S3 Table). We used 20 μl reaction volume containing 0.5 μM each primer, 2 mM MgCl_2,_ 200 μM dNTPs, 1X Taq buffer and 1 U Taq DNA polymerase (Invitrogen). Cycling conditions included an initial denaturing step at 94°C for 1 min, followed by 30 cycles of 94°C for 1 min, 50 or 55°C (according to respective Tm) for 1 min, and 72°C for 3 min, with a final extension step at 72°C for 7 min. Products were separated by electrophoresis in 1% agarose gels and then, cloned and sequenced. As controls, for PCR amplification of DNA we omitted the DNA in the reaction mixture, and for RT-PCR we used DNAse-treated RNA as template. For RT-PCR assays, 100 ng of cDNA, 0.15 μM of each primer (S3 Table) and the KAPA SYBR FAST PCR Master Mix (Kapa Biosystems) were used in a StepOne^TM^ Real-Time PCR System (Applied Biosystem). Data from three independent cDNA preparations were analyzed using the 2^−ΔΔ*Ct*^ method with *ribosomal 40s* S2 protein as house-keeping gene.

### Production of α-EhNPC1 and α-EhNPC2 antibodies

The DEQPMYDKDGQYVPVEKRLE polypeptide from EhNPC1 (776 to 785 amino acids) was synthesized together with the KLH (Keyhole Limpet Hemocyanin) tag to increase its immunogenicity (GenScript). New Zeland rabbits (already-existing collection) were immunized first with100 μg of this polypeptide resuspended in Titermax Gold adjuvant (1:1) (Sigma) and then, with two more weekly doses of 50 μg each, to generate the α-EhNPC1 antibody.

The *Ehnpc2a* and *Ehnpc2b* full-length genes were PCR-amplified using cDNA as template and specific primers, which introduced unique *BamHI* and *SalI* restriction enzyme sites, in the sense and antisense primers, respectively (underlined in S3 Table). Genes were cloned into the *pGEX6P-1* plasmid to generate *pGEX6P-Ehnpc2a* and *pGEX6P-Ehnpc2b* constructs. *E*. *coli* pLys-S bacteria were transformed with the plasmids, then, recombinant proteins were obtained by 0.1 mM IPTG induction. Recombinant proteins were electro-eluted and purified by size exclusion chromatography using a PD-10 column (GE Healthcare). Then, 60 μg of each purified recombinant protein (rEhNCP2a and rEhNPC2b) were emulsified in Titer-Max Gold adjuvant (Sigma) and subcutaneously and intramuscularly inoculated in Wistar rats (already-existing collection) and BALB/C mice (already-existing collection), respectively. Two more dose**s** (30 μg) of each protein were injected at 20 day intervals followed by bleeding to obtain antibodies. In all experiments, we used the rat α-EhNPC2a antibody (α-EhNPC2) as pan antibody, because it detected both EhNPC2a and EhNPC2b recombinant proteins. Pre-immune serum was obtained before immunizations.

### Cell fractionation and western and dot blot assays

Trophozoite extracts (30 μg) were centrifuged 10 min at 13,000xg, and, soluble (supernatant) and insoluble (pellet) fractions were obtained. Samples were separated by 10 or 12% sodium dodecyl sulfate polyacrylamide gel electrophoresis (SDS-PAGE), transferred to nitrocellulose membranes and probed with rabbit α-EhNPC1 (1:3000) or rat α-EhNPC2 (1:3000) or rabbit α-EhADH (1:500) [24] or rabbit α-EhRab7A (1:200) (kindly provided by Dr. Tomoyoshi Nozaki, National Institute of Infectious Diseases, Tokyo, Japan) [36] or rabbit α-EhCP112 (1:3000) [48] or rabbit α-SERCA (1:200) [32] or mouse α-actin (1:200) [49] antibodies. Membranes were washed and incubated with the species-specific HRP-labeled secondary antibodies (Zymed; 1:10000), and developed with ECL Prime detection reagent (GE-Healthcare). Pre-immune sera were used as controls. For dot blots analysis, samples were prepared as described [26], dripped on nitrocellulose membranes and treated with rabbit α-cholesterol antibodies (1:100). (Cloud Clone Co) followed by HRP-labeled secondary antibodies.

### Laser confocal microscopy experiments

Trophozoites (grown in coverslips) in basal conditions or after phagocytosis were fixed with 4% paraformaldehyde (PFA) at 37°C for 1 h, permeabilized with 0.2% Triton X-100 and blocked with 10% fetal bovine serum (FBS) in PBS. Then, preparations were incubated at 37°C for 1 h with α-EhNPC1 (1:100) or α-EhNPC2 (1:100) or α-EhADH (1:500) or α-EhRab7A (1:500) or α-Gal/GalNAc lectin (1:50) (kindly provided by Dr. W. Petri, University of Virginia, Charlottesville, USA) [50] or α-LBPA (1:30) [23, 26] or α-EhSERCA (1:1000) antibodies; followed by extensive washing and incubation for 1 h with species-specific FITC-, TRITC- or Cy5- labeled secondary antibodies (Zymed; 1:100) as appropriate. In some cases, nuclei were counterstained with propidium iodide (0.1 μg/ml) (Sigma) for 5 min. For cholesterol detection, cells were stained with 250 μg/ml filipin (Sigma). For acidic vacuoles detection, fixed trophozoites were incubated with 2 μg/ml of Lysotracker (Molecular Probes) for 2 h at 37°C. We also performed experiments using 12 h ABS-starved trophozoites (incubated in TYI medium), cultured on coverslips, and challenged with 100 μl of ABS for 0.5 to 7 min at 37°C, then, samples were processed as above. All preparations were preserved using Vecta Shield antifade reagent (Vector), examined using a Carl Zeiss LMS 700 confocal microscope and processed with ZEN 2009 Light Edition Software (Zeiss). To evaluate the co-localization between molecules, Pearson coefficients (PC) were obtained from at least 15 confocal independent images (laser sections: 0.5 μm) using the ImageJ 1.45v software and the JACoP plugin [51].

### Transmission electron microscopy (TEM)

For immunogold-labeling experiments, trophozoites in basal conditions were fixed with 4% PFA and 0.5% glutaraldehyde in PBS for 1 h at room temperature (RT). Samples were embedded in LR White resin (London Resin Co) and polymerized under UV at 4°C for 48 h. Thin sections (60 nm) were mounted on formvar-covered nickel grids followed by overnight (ON) incubation with α-EhNPC1 or α-EhNPC2 antibodies (1:20) and, then, incubated ON, with the respective secondary antibodies (1:60) conjugated to 20 and 10 nm gold particles, respectively (Ted Pella Inc.). Thin sections were contrasted with uranyl acetate and lead citrate and observed with a Jeol JEM-1011 transmission electron microscope. Number of gold particles was counted in 12 independent images of 10 μm^2^ each.

### Cholesterol quantification

Trophozoites (5x10^5^) were re-suspended in a mixture of chloroform: isopropanol:Nonidet P40 (7:11:1) and centrifuged at 13,000xg for 10 min. The organic phase was collected and dried at 50°C. Lipid samples were processed according to the Cholesterol Quantitation kit (Sigma)

### Secretion assay

Trophozoites were grown in TYI medium for 12 h and incubated with 100 μl of ABS for 0.5, 5 and 15 min at 37°C. Then, medium was collected in the presence of 1 mg/ml of E64 (Sigma) and centrifuged at 13,000xg for 10 min to obtain the secreted molecules in the supernatant fraction. To obtain the trophozoite extracts, cells were washed and lysed with proteases inhibitors as reported [52]. Samples were submitted to 12% SDS-PAGE and western blot assays using α-EhNPC1, or α-EhNPC2, or α-cholesterol, or α-EhCP112 or α-actin antibodies as described above.

### Immunoprecipitation assays

Trophozoites were lysed in the presence of 10 mM Tris-HCl, 50 mM NaCl and protease inhibitors by freeze-thawing cycles and vortexing. Immunoprecipitation assays were performed using 200 μl of protein G-agarose (Invitrogen) and α-EhNPC1 or α-EhNPC2 antibodies as described [53]. Immunoprecipitates were analyzed by SDS-PAGE and western blot assays using α-EhNPC1 or α-EhNPC2 or α-EhADH or α-EhRab7A or α-EhSERCA or α-cholesterol antibodies, as described [24].

### Phagocytosis assays

Trophozoites were incubated with human erythrocytes (already-existing collection) (1:25) at 37°C (for phagocytosis assays) for 5 to 60 min. For pulse-chase experiments, incubation was carried out for 5 min at 37°C. Then, preparations were quickly washed three times with TYI-water (1:1) to remove the adhered and non-ingested erythrocytes. Adhered and non-ingested erythrocytes were lysed by incubation in distilled water for 10 min at RT. Then, trophozoites and ingested erythrocytes were lysed using absolute formic acid and the hemoglobin was quantified by spectrophotometry at 400 nm [54]. In some experiments ingested erythrocytes were stained by Novikoff solution [55] and samples were observed through the light microscope (Axiolab, Zeiss).

### *In vivo* virulence assays

Mice C57BL/6 strain (already-existing collection, 8–12 week old in a weight range of 22–30 g each), were anesthetized by an intraperitoneal injection of 25 mg/ml of ketamine and 2.5 mg/ml of xylazine in PBS. Then, 10^6^ trophozoites were anally inoculated (with previously glycerol lubrication) in five mice per condition. As a control, mice were inoculated with PBS. After 30 min, laparotomy was performed, a cannula was inserted through a small incision in the proximal colon and the colon was washed extensively with PBS. After this, using the same cannula, Evans blue dye was administrated into the colon and mice were left at RT for 15 min. Then, anesthetized mice were euthanized by cervical dislocation. Subsequently, the colon was flushed with abundant PBS to remove the dye, followed by washing with 1 mM N-acetylcysteine to remove dye from mucus. Colon was dissected, weighted and the leaked dye was extracted with gentle shaking in 2 ml dimethyl formamide and spectrophotometrically measured at 620 nm. Permeability was calculated as OD_620_ per g tissue.

### Hematoxylin and eosin staining

Colon samples were fixed in 10% phosphate-buffered formalin and processed for conventional embedding paraffin, then, 4–6 μm sections were stained with hematoxylin and eosin [56]. Samples were analyzed using a light microscope (Axiolab, Zeiss).

### Plasmid construction for silencing experiments

The first 400 or 420 bp from the 5′-end of *Ehnpc1* and *Ehnpc2a* genes, respectively, were PCR amplified using specific primers (S3 Table) and cloned into the vector psAP-2 downstream of the 5′ upstream segment (473 bp) of the *ap-a* gene [40].

### Transfection assays

Trophozoites of clone G3 were transfected as described [57]. Briefly, G3 trophozoites were cultured in 35-mm Petri dishes and transfected with 20 μg of the corresponding plasmid: psAP-2-*Ehnpc1* (1–400 bp) or psAP-2-*Ehnpc2a* (1–420 bp) containing gene fragments, using SuperFect (Qiagen) reagent. The transfected parasites were incubated for 48 h at 37°C, selected by 4 μg/ml of G-418 (Sigma-Aldrich) and maintained as a stable cell line. Then, *Ehnpc1* and *Ehnpc2a* silencing was confirmed by RT-qPCR, western blot analysis and immunofluorescence.

### Migration assays

Serum-starved (3 h) trophozoites (7.5x10^4^) were placed in the upper chamber of transwell inserts (5 μm pore size, 24 well, Costar) and 500 μl of ABS were added to the lower chamber. Trophozoites were then incubated for 3 h at 37°C. At the end of the incubation, the inserts and media were removed, and trophozoite migration was determined by counting the number of trophozoites that were attached to the lower chamber of the well.

### Statistical analysis

Values of all assays were expressed as mean ± standard error of three independent experiments, each in duplicate. Statistical analyzes were carried out using the GraphPad Prism V 5.01 software by Anova or Studentˈs test.

### Ethic Statements

The Institutional Animal Care and Use Committee (IACUC) ethics committee reviewed and approved the animal care and use of mice, rabbits and rats to produce antibodies and mice used in virulence *in vivo* experiments (Protocol Number 0313–06) by the document CICUAL 001, in which is specified that our institute fulfils the NOM-062-ZOO-1999 that deals with the Technical Specifications for Production, Care and Use of Laboratory Animals given by the General Direction of Animal Health of the Minister of Agriculture (SAGARPA), that verify the fulfil of the international regulations/guidelines for the use and care of animals used in laboratory and has verified and approved the animal care at CINVESTAV (Verification Approval Number: BOO.02.03.02.01.908).

## Supporting Information

S1 Fig Inmunodetection of EhNPC2 proteins. Western blot assays of rEhNPC2a and rEhNPC2b recombinant proteins immunodetected with rat α-EhNPC2a antibodies. Trophozoites lysates (ET) were used as a positive control.
(TIF)

S1 Table Sequence analysis of NPC1 in orthologues organisms (DOCX)

S2 Table Sequence analysis of NPC2 in orthologues organisms (DOCX)

S3 Table Oligonucleotides sequences. *BamHI* and *SalI* restriction enzyme sites are underlined in forward and reverser primers, respectively.
(DOCX)
