# Supplementary figures and images for: EhNPC1 and EhNPC2 Proteins Participate in Trafficking of Exogenous Cholesterol in Entamoeba histolytica Trophozoites: Relevance for Phagocytosis
Source: PLoS Pathog. 2016 Dec 21;12(12):e1006089. doi: 10.1371/journal.ppat.1006089 (PMC5176366; doi:10.1371/journal.ppat.1006089)

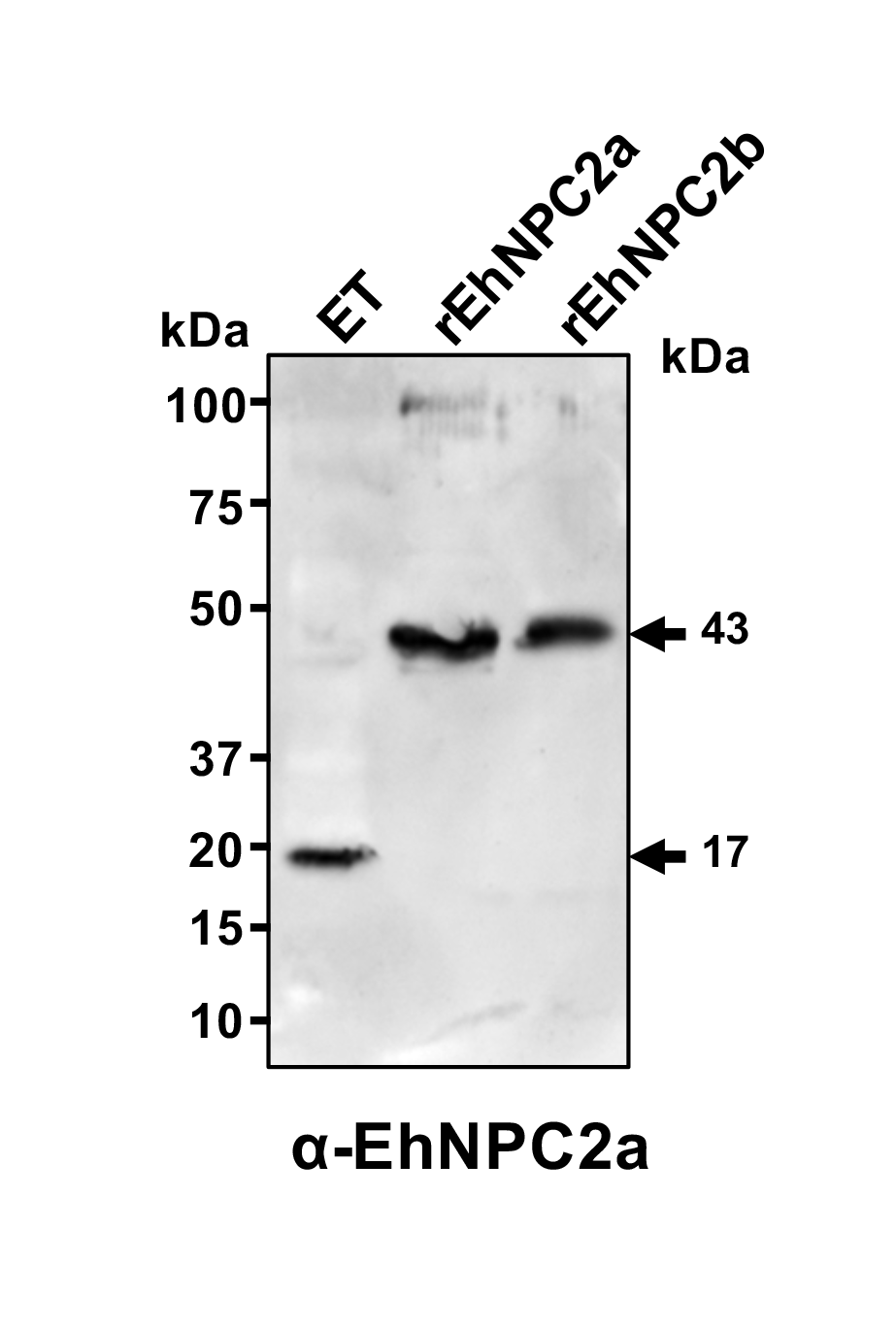

Supplement: S1 Fig — Western blot assays of rEhNPC2a and rEhNPC2b recombinant proteins immunodetected with rat α-EhNPC2a antibodies. Trophozoites lysates (ET) were used as a positive control. (TIF) [file ppat.1006089.s001.tif]
