# Supplementary material for: EhNPC1 and EhNPC2 Proteins Participate in Trafficking of Exogenous Cholesterol in Entamoeba histolytica Trophozoites: Relevance for Phagocytosis
Source: PLoS Pathog. 2016 Dec 21;12(12):e1006089. doi: 10.1371/journal.ppat.1006089 (PMC5176366; doi:10.1371/journal.ppat.1006089)
Supplement: S1 Table — (DOCX) [file ppat.1006089.s002.docx]

**Table S1. Sequence analysis of NPC1 in orthologues organisms**

| **Organism** | **Protein name** | **Accession number (KEGG)** | **Identity (%)** | **E value** |
| --- | --- | --- | --- | --- |
| *Saccharomyces cerevisiae* | NCR1p | sce:YPL006W | 27.8 | 5e-98 |
| *Candida albicans* | NCR1 | cal:CaO19.7242 | 26.9 | 3e-116 |
| *Aspergillus fumigatus* | NCR1 | afm:AFUA_6G09980 | 28.8 | 7e-141 |
| *Dictyostelium discoideum* | NPC | ddi:DDB_G0269158 | 34.5 | 0.0 |
| *Homo sapiens* | NPC1 | hsa:4864 | 31.7 | 2e-150 |
| *Homo sapiens* | NPC1-like 1 | hsa:29881 | 28.9 | 6e-121 |
| *Drosophila melanogaster* | NPC1a | dme:Dmel_CG5722 | 30.7 | 2e-157 |
| *Aedes aegypti* | NPC1 | aag:AaeL_AAEL003325 | 35.0 | 9e-136 |
| *Bos taurus* | NPC1 | bta:286772 | 31.8 | 2e-150 |
| *Caenorhabditis elegans* | NCR1 | cel:CELE_F02E8.6 | 24.2 | 5e-75 |
| *Trichomonas vaginalis* | Cation efflux family protein | tva:TVAG_237080 | 30.1 | 4e-05 |
| *Toxoplasma gondii* | Patched family domain containing protein | tgo:TGME49_085470 | 20.1 | 7e-22 |
| *Leishmania major* | Hypothetical protein | lma:LMJF_28_0340 | 19.7 | 4.5 |
