# Supplementary material for: EhNPC1 and EhNPC2 Proteins Participate in Trafficking of Exogenous Cholesterol in Entamoeba histolytica Trophozoites: Relevance for Phagocytosis
Source: PLoS Pathog. 2016 Dec 21;12(12):e1006089. doi: 10.1371/journal.ppat.1006089 (PMC5176366; doi:10.1371/journal.ppat.1006089)
Supplement: S2 Table — (DOCX) [file ppat.1006089.s003.docx]

**Table S2. Sequence analysis of NPC2 in orthologues organisms**

| **Organisms** | **Protein name** | **Accession number (KEGG)** | **Identity (%)** | | | **E value** | | |
| --- | --- | --- | --- | --- | --- | --- | --- | --- |
|  |  |  | **2a** | **2b** | **2a** | | **2b** |  |
| *Entamoeba histolytica* | EhNPC2a | Ehi_068260 | 100 | 29.41 | 100 | | 1.3e-15 |  |
| *Entamoeba histolytica* | EhNPC2b | Ehi_188770 | 29.41 | 100 | 1.3e-15 | | 100 |  |
| *Dictyostelium discoideum* | Hypothetical protein | DDB_G0270454 | 25.98 | 24 | 4.1e-09 | | 8.8e-10 |  |
| *Caenorhabditis elegans* | Hypothetical protein | CELE_F30H5.3 | 18.55 | 17.83 | 4.4e-05 | | 1.1e-05 |  |
| *Drosophila melanogaster* | Npc2a | Dmel_CG7291 | 13.45 | 11.20 | 0.0016 | | 0.00075 |  |
|  | Npc2b | Dmel_CG3153 | 18.80 | 14.07 | 0.063 | | 0.0055 |  |
|  | Npc2d | Dmel_CG12813 | 19.70 | 17.27 | 0.05 | | 0.014 |  |
|  | Npc2e | Dmel_CG31410 | 14.17 | 14.07 | 0.05 | | 0.00013 |  |
| *Homo sapiens* | Npc2 | hsa:10577 | 14.63 | 18.46 | 0.0091 | | 5.2e-05 |  |
| *Bos taurus* | Npc2 | bta:280815 | 15.45 | 16.92 | 0.0018 | | 0.00088 |  |
| *Aedes aegypti* | Npc2 | AaeL_AAEL006854 | 13.49 | 17.56 | 0.0027 | | 0.14 |  |
| *Saccharomyces cerevisiae* | Npc2p | YDL046W | 25.93 | 21.58 | 1.5e-07 | | 6.9e-06 |  |
| *Candida albicans* | Similar Npc2p | CaO19.10736 | 24.09 | 20.42 | 1.2e-08 | | 2e-08 |  |
