# Supplementary material for: EhNPC1 and EhNPC2 Proteins Participate in Trafficking of Exogenous Cholesterol in Entamoeba histolytica Trophozoites: Relevance for Phagocytosis
Source: PLoS Pathog. 2016 Dec 21;12(12):e1006089. doi: 10.1371/journal.ppat.1006089 (PMC5176366; doi:10.1371/journal.ppat.1006089)
Supplement: S3 Table — BamHI and SalI restriction enzyme sites are underlined in forward and reverser primers, respectively. (DOCX) [file ppat.1006089.s004.docx]

**Table S3. Oligonucleotides sequences.** *BamH I* and *Sal I* restriction enzyme sites are underlined in forward and reverser primers, respectively.

| **Complete gene** | **Sequence primer** | | **Amplicon size (bp)** |
| --- | --- | --- | --- |
| *Ehnpc1* | F | GCGGATCCATGTTTGTTTTCTTCTTCTTGATAAGTGTTGTAATTAGTATTAC | 4020 |
|  | R | CCGTCGACCATTCAAAGTTTTGATATTCTCCTTTTGTACTCTT |  |
| *Ehnpc2a* | F | GCGGATCCATGCCATTGGTTAACCCATTATTTTTTTTATTACTTATTTCATTC | 441 |
|  | R | CCGTCGACCTATTTAACTAATACCGGACCAAATTCTGAACAACTGAATAGAGTGC |  |
| *Ehnpc2b* | F | GCGGATCCATGTTTGTGTTATTCATACTATTCACTACTCTTTTTGCA | 426 |
|  | R | CCGTCGACTTATTTCATATCAAGCATAAATCCTAAACAAGTGATATTATAGTCACC |  |
| **qRT-PCR** | **Sequence primer** | | **Amplicon size (bp)** |
| *Ehnpc1* | F | ATTTACCTCTGCTGGATTCTTCTC | 164 |
|  | R | GGAACATACTGACCATCTTTATCG |  |
| *Ehnpc2a* | F | CAGTGAGTGGTTTAGCATCAG | 131 |
|  | R | CCTTTGAAAGTTGTGGAACAG |  |
| *Ehnpc2b* | F | CTCCATTTGTCTACCTGAACC | 232 |
|  | R | CTGGACATCTAACTACTGTTCC |  |
| *ribosomal s2* | F | ATTCGGAAATAGAAGAGGAGG | 104 |
|  | R | ACTATTCTTCCAAGCTTGGT |  |
| **Silencing genes** |  | **Sequence primer** | **Amplicon size (bp)** |
| *Ehnpc1* | F | AGCTAGGCCTATGTTTGTTTTCTTCTTCTTGAT | **400** |
|  | R | GCATGAGCTCTCATCAATTTCAGTCCATCTTG |  |
| *Ehnpc2a* | F | AGCTAGGCCTATGTTTGTGTTATTCATACTATTCAC | **420** |
|  | R | GCATGAGCTCAACAAGTGATATTATAGTCACCG |  |
